# Comparative outcomes of focal laser versus 360-degree laser in vitrectomy for retinal detachment: a systematic review and meta-analysis

**DOI:** 10.1186/s40942-025-00790-2

**Published:** 2026-01-09

**Authors:** Valdez Melo dos Anjos Filho, Natalia Garcia Gaban, Sara Hira, Maria Antônia Torres Arteche, Mateus Pimenta Arruda, Bernardo Bolzani Bach

**Affiliations:** 1 Penido Burnier Institute, Campinas, Brazil; 2 Federal University of Vales do Jequitinhonha e Mucuri, Teófilo Otoni, Brazil; 3 Federal University of Mato Grosso do Sul, Campo Grande, Brazil; 4 FMH College of Medicine & Dentistry, Lahore, Pakistan; 5 Federal University of Rio Grande do Sul, Porto Alegre, Brazil; 6 Federal University of São Paulo, São Paulo, Brazil; 7 University of Iowa, Iowa city, USA

**Keywords:** Retina, Lasers, Retinal detachment, Vitrectomy

## Abstract

**Purpose:**

The choice of technique for primary repair in rhegmatogenous retinal detachment (RRD) is of extreme importance for the visual prognosis. However, no study has been able to statistically determine the role of 360° endolaser retinopexy compared to focal retinopexy during intraoperative pars plana vitrectomy (PPV), emphasizing their associated benefits and complications. Therefore, this meta-analysis aimed to compare whether performing 360° laser would result in higher rates of Single Surgery Anatomical Success (SSAS) (primary outcome), and lower rates of postoperative epiretinal membrane (ERM) and macular edema (ME) (secondary safety outcomes), and Best-Corrected Visual Acuity (BCVA) (secondary functional outcome), compared to focal endolaser.

**Methods:**

The primary outcome was Single Surgery Anatomical Success (SSAS). The secondary outcomes included postoperative Best-Corrected Visual Acuity (BCVA) (functional), and the incidence of Epiretinal Membrane (ERM) and macular edema (safety). Eligibility criteria encompassed randomized controlled trials (RCTs) and nonrandomized cohort studies that compared 360° laser retinopexy to focal retinopexy during pars plana vitrectomy for rhegmatogenous retinal detachment (RRD). Studies published up to the search date were included. We systematically searched PubMed, Scopus, and Cochrane from May 2024 to November 2025, following the PRISMA guidelines. The primary outcomes measured were Single Surgery Anatomical Success (SSAS) and postoperative Best-Corrected Visual Acuity (BCVA). Secondary outcomes included the incidence of Epiretinal Membrane (ERM) and macular edema. We performed a meta-analysis using the random-effects model and assessed heterogeneity using the I² statistic. We evaluated the risk of bias using the Cochrane Risk of Bias tool for RCTs and the ROBINS-I tool for nonrandomized studies. We conducted a pre-specified subgroup analysis based on the study design. Finally, we assessed the Certainty of Evidence using the GRADE system.

**Results:**

Pooled analysis of 4.320 eyes and ten studies revealed that the 360-degree laser group was associated with a higher incidence of Single Surgery Anatomical Success (SSAS) (OR = 0.79; 95% CI 0.65–0.95). In contrast, the combined analysis of six articles revealed that there was no statistically significant difference in Best-Corrected Visual Acuity (BCVA) between the groups (MD = 0.06; 95% CI -0.01–0.13). The incidence of epiretinal membrane formation epiretinal membrane (ERM) was lower at the focal laser group (OR = 0.98, 95% CI 0.67–1.44). Finally, the combined analysis revealed no significant difference in the rate of macular edema between the groups (OR = 1.04; 95% CI 0.61–1.79).

**Conclusion:**

Compared with focal laser therapy, 360° criterion laser prophylaxis during PPV was superior in terms of single surgery anatomical success (SSAS) in the treatment of rhegmatogenous retinal detachment (RRD). However, we must understand these findings with caution due to the broad inclusion of various study designs, particularly nonrandomized retrospective cohorts. Although this approach provided a broader evidence base, it introduces potential bias, as highlighted by the quality assessment. Furthermore, limitations in the randomization process observed in one of the two included randomized controlled trials (RCTs) slightly weaken the overall statistical support for clinical application. The absence of any statistically significant differences in functional outcomes Best-Corrected Visual Acuity (BCVA) or the development of complications between the methods indicates that, currently, the choice of treatment should be highly individualized based on the patient’s characteristics and clinical situation. Better-designed RCTs are necessary to support the superior anatomical success observed and to establish a standard of care.

**PROSPERO registration ID:**

CRD42024568314.

**Supplementary Information:**

The online version contains supplementary material available at 10.1186/s40942-025-00790-2.

## Introduction

Rhegmatogenous retinal detachment (RRD) is a serious vision-threatening condition that affects approximately 10 to 18 people per 100,000 people per year in the United States [1, 2]. 

Retinal detachment involves the separation of the retinal pigment epithelium (RPE) from the neurosensory retina. This condition results from a full-thickness retinal rupture, which allows liquefied vitreous to enter the subretinal space [3]. This potential space exists because the neurosensory retina and the RPE derive from the same embryonic layers. Furthermore, although the layers are adjacent, they lack strong anatomical junction cells that form adhesions [3]. 

Surgeons must repair an acute, symptomatic retinal detachment as quickly as possible (preferably within one to two days). The need for immediate intervention is particularly high when the detachment involves the macula. Overall, these cases constitute one of the most common ophthalmologic emergencies [3, 4].

There are several options for treating RRDs based on surgeon experience, patient age, lens status, the presence of proliferative vitreoretinopathy, the number and position of the tears and the presence of other associated conditions. However, the use of lasers or cryotherapy to barrage tears is a common step among all the techniques [2]. 

In recent years, pars plan vitrectomy (PPV) has become the most commonly used treatment for RRDs, especially in the United States, where it represents approximately 70% of procedures [5]. In Germany, for example, physicians/clinicians observed a significant decrease in the proportion of initial surgeries using scleral buckle compared to vitrectomy [4]. Furthermore, surgeons/techniques showed improvements, such as sutureless trocar vitrectomy, and clinicians decreased the primary use of silicone oil as a tamponade agent [4]. 

The anatomical and visual success of surgery requires careful preoperative assessment, which is associated with the closure of retinal ruptures and patient cooperation in the postoperative period, in addition to intrinsic ocular factors, with results varying between 64% and 91% primary success [5, 6]. 

During surgery, some surgeons recommend the addition of a 360-degree barrage with two or three rows of laser in the retinal periphery to supplement the laser around the retinal breaks. The barrage would create a chorioretinal scar capable of increasing retinal adhesion and, consequently, single surgery success. However, there is still no consensus among surgeons regarding this technique [4]. Warren et al. reported lower final anatomic success rates and poorer visual acuity outcomes when a 360-degree laser was used when adjusted for case complexity. On the other hand, Peters et al. reported no difference in surgical outcomes or complication rates, and Dirani et al. reported a significant reduction in the odds of postoperative retinal redetachment with a 360-degree laser in eyes with uncomplicated primary RRD [7, 8]. 

To address this inconsistency in the literature, we performed a meta-analysis of randomized and retrospective studies comparing single surgery anatomical success (SSAS) and postoperative best corrected visual acuity (BCVA) between patients who underwent PPV and those who received 360 lasers and patients who received only focal lasers around retinal tears.

Researchers published a similar meta-analysis in 2021, analyzing the same outcomes. However, new studies have emerged in recent years that allow for updated results. Additionally, a study comparing laser 360 versus scleral buckling was included, which deviates from our goal of analyzing differences between laser techniques in patients who underwent PPV [9]. 

## Methods

### Eligibility criteria

We utilized the PICOS framework to define our eligibility criteria, focusing on the following elements:

P (Patient Population): We focused on patients diagnosed with rhegmatogenous retinal detachment (RRD), including those with single or multiple retinal breaks. We permitted the inclusion of studies featuring minor associated clinical characteristics, such as phakic or pseudophakic status, but excluded eyes with Proliferative Vitreoretinopathy (PVR) grade C or higher, or RRDs related to trauma, endophthalmitis, or retinectomy. Studies involving choroidal detachment were also excluded, as this variable acts as a significant confounder.

I (Intervention) and C (Control): The selected studies compared intraoperative prophylactic 360-degree laser retinopexy (Intervention) versus focal laser retinopexy (Control) in patients who underwent pars plana vitrectomy (PPV). Studies comparing the 360° laser against other surgical techniques, such as scleral buckling, were excluded to ensure a direct comparison of laser retinopexy techniques during PPV.

O (Outcome Reporting): Studies were required to report at least one set of efficacy or safety outcomes (SSAS, BCVA, ERM, or Macular Edema) for each treatment arm.

S (Study Design & Publication): Eligibility criteria encompassed Randomized Controlled Trials (RCTs) and nonrandomized cohort studies. We included only peer-reviewed full-text articles to ensure the credibility and rigor of the selected literature. We required a minimum sample size of 25 patients/eyes to ensure a sufficient level of statistical power and meaningful contribution to the pooled analysis.

### Search strategy and data extraction

We systematically searched PubMed (MEDLINE), Scopus, and the Cochrane Central Register of Controlled Trials from May to June 2024 with the following search terms: ‘Retinal detachment‘[Mesh], ‘Vitrectomy‘[Mesh], ‘Panretinal’, ‘Circumferential’, ‘360’, ‘360°’, ‘Three hundred and sixty’, ‘360 degree’, ‘360-laser’, ‘Photocoagulation‘[Mesh], ‘Laser‘[Mesh], ‘Retinopexy’, ‘Focal’, and ‘Localized’.

We also manually searched the references from all included studies, systematic reviews, and previous meta-analyses for any additional studies. Two authors (S.H. and V.M.) independently extracted the data according to predefined search criteria. We used the screening tool Zotero to manage the retrieved results, perform duplicate removal, and facilitate independent screening of titles and abstracts based on the predefined eligibility criteria. Discrepancies were resolved through consensus between the authors. We registered the protocol for this meta-analysis on PROSPERO on July 11, 2024, under protocol #CRD42024568314.

### Endpoints

Primary Outcome (Efficacy): The primary outcome of interest was the incidence of Single Surgery Anatomical Success (SSAS).

Secondary Outcomes (Functional and Safety): Secondary outcomes included postoperative Best-Corrected Visual Acuity (BCVA) (functional) and the incidence rates of Epiretinal Membrane (ERM) formation and macular edema (safety).

### Quality assessment

We evaluated the risk of bias in randomized studies via version 2 of the Cochrane risk of bias assessment tool (RoB2- Table 1) [10]. Nonrandomized studies were assessed with the Risk of Bias in Nonrandomized Studies of Interventions tool (ROBINS-I – Table 2) [11].

**Table 1:** Table with the risk of bias for the two randomized studies, made with RoB 2. Risk of bias summary for randomized studies (RoB 2)

Study	Bias from randomization process	Bias due to deviations from intended interventions	Bias due to missing outcome data	Bias in measurement of the outcomes	Bias in selection of the reported result	Overall risk of bias
BILGINS 2018	Some concerns	Low	Low	Low	Low	Some concerns
LOUIDICE 2019	Low	Low	Low	Low	Low	Low

**Table 2:** Table with the risk of bias for the eight non-randomized studies, made with ROBINS-I. Risk of bias summary for nonrandomized studies (ROBINS-I)

Study	Bias due to confounding	Bias in selection of participants	Bias in classification of interventions	Bias due to deviations from intended interventions	Bias due to missing data	Bias in measurement of outcomes	Bias in selection of the reported result	Overall risk of bias judgment
Mathai 2002	Moderate	Low	Low	Low	Low	Low	Low	Moderate
Iwase 2013	Serious	Low	Low	Low	Low	Low	Moderate	Serious
Dirani 2019	Moderate	Low	Low	Low	Low	Low	Moderate	Moderate
Zheng 2023	Serious	Moderate	Low	Low	Low	Low	Moderate	Serious
Ryoo 2023	Serious	Low	Low	Low	Low	Low	Moderate	Serious
Wang 2019	Serious	Low	Low	Low	Low	Low	Low	Serious
Peters 2022	Serious	Low	Low	Low	Low	Low	Low	Serious
Cristescu 2023	Serious	Low	Low	Low	Low	Low	Moderate	Serious

Two independent authors completed the risk of bias assessment (M.P. and V.M.). Disagreements were resolved through a consensus after a discussion of the reasons for discrepancy with the participation of a third independent author (B.B.). We assessed publication bias via funnel plot analysis of point estimates in relation to study weights.

### Statistical analysis

We performed and wrote this systematic review and meta-analysis in accordance with recommendations from the Cochrane Collaboration Handbook for Systematic Review of Interventions and the Preferred Reporting Items for Systematic Reviews and Meta-Analysis (PRISMA) Statement.

Synthesis Methods: We used the random-effects model (DerSimonian and Laird) for outcomes with significant heterogeneity (I² > 25%), and the fixed-effects model for low heterogeneity. We used odds ratios (ORs) for binary outcomes and mean differences (MDs) for continuous outcomes, calculated with 95% confidence intervals (CIs).

Data Collection Process: We estimated standard deviations from other statistics, such as p values or confidence intervals, via the Review Manager Calculator when they were not directly available in the source study.

Certainty Assessment Methodology (GRADE): Finally, we assessed the Certainty of Evidence for all primary and secondary outcomes using the Grading of Recommendations Assessment, Development and Evaluation (GRADE) system (Table 3) [12]. This evaluation considered the risk of bias, inconsistency (I²), imprecision, indirectness, and publication bias. We also performed sensitivity analyses by removing each individual study from the outcome assessment. (Cochrane Center, The Cochrane Collaboration, Denmark).

**Table 3:** “GRADE System” (an acronym for grading of Recommendations, assessment, development and Evaluation) is a transparent and systematic method widely used to: assess the certainty of evidence (or quality of evidence) and determine the strength of recommendations in healthcare. GRADE System

Outcome	No. of Studies / Participants	Effect (Pooled Results)	Certainty of Evidence	Comments
Single Surgery Anatomical Success (SSAS)	10 studies / 4,320 eyes	OR = 0.79 (95% CI: 0.65 to 0.95).	LOW	Risk of Bias: Serious. Majority of studies were nonrandomized (7/10), with serious/moderate risk of selection/confounding bias (ROBINS-I) Inconsistency: Low heterogeneity (I² = 22%)
Best-Corrected Visual Acuity (BCVA)	6 studies / 3,358 eyes	MD = 0.06 (95% CI: -0.01 to 0.13).	VERY LOW	Inconsistency/Heterogeneity: Very High (I² = 79%), indicating significant variation in results. Imprecision: The 95% CI crosses the no-effect boundary (0), resulting in a non-statistically significant difference.
Epiretinal Membrane (ERM) Formation	9 studies / 4,227 eyes	OR = 0.98 (95% CI: 0.67 to 1.44).	LOW	Risk of Bias: High (due to retrospective studies) Inconsistency/Heterogeneity: Moderate (I² = 47%) Imprecision: The 95% CI crosses the no-effect boundary (1.00), resulting in a non-statistically significant difference
Macular Edema	5 studies / 3,153 eyes	OR = 1.04 (95% CI: 0.61 to 1.79).	LOW	Risk of Bias: High. Inconsistency/Heterogeneity: Moderate (i² = 36%) Imprecision: The 95% CI is wide and crosses the no-effect boundary (1.00), indicating a non-significant difference

We identified study design (Randomized Controlled Trial vs. Nonrandomized Cohort) as a prespecified factor of potential heterogeneity and performed a subgroup analysis based on this criterion. Furthermore, if significant heterogeneity (I² > 50%) persisted for a primary outcome, we planned to conduct a meta-regression analysis to formally investigate potential sources of heterogeneity. The covariates considered in this analysis would include mean patient age, proportion of eyes with macula-off detachment, and mean number of retinal breaks, as these factors commonly influence RRD outcomes and may introduce confounding between the laser groups. This approach would aim to quantify how much of the observed variability in effect estimates the specified covariates explain.

## Results

### Study selection and baseline characteristics

**Fig. 1:**
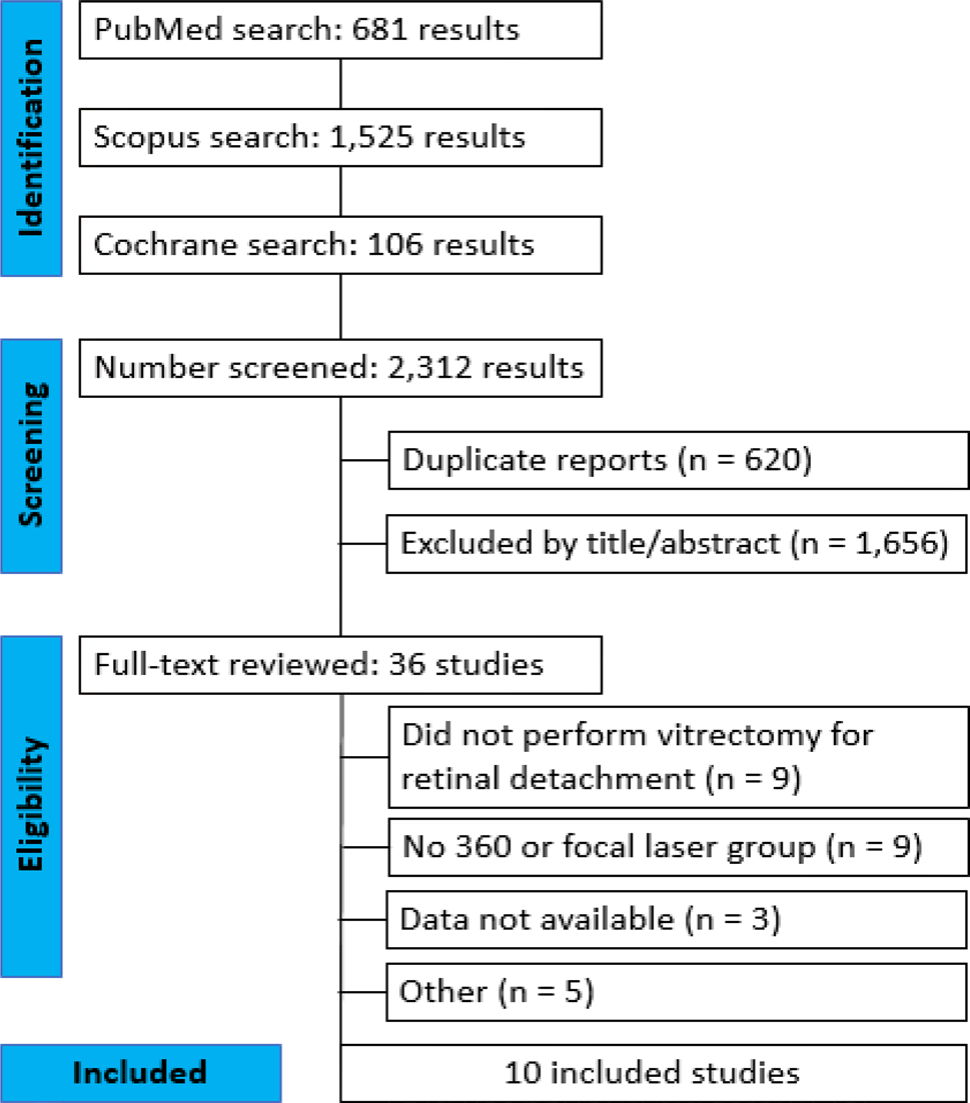
PRISMA flow diagram of study screening and selection

The search strategy identified a total of 2,312 abstracts or manuscripts, as illustrated in Fig. 1. After the removal of duplicate records and unrelated studies based on the title and abstract criteria, we fully reviewed 36 studies according to the inclusion and exclusion criteria. Of these, we excluded 26. Finally, 10 studies were included in this systematic review and meta-analysis.

A non-overlapping population of 4,320 eyes was included, of which 1,447 (33.49%) received 360-degree laser retinopexy. The baseline characteristics of the selected studies are in Table 4.

**Table 4:**
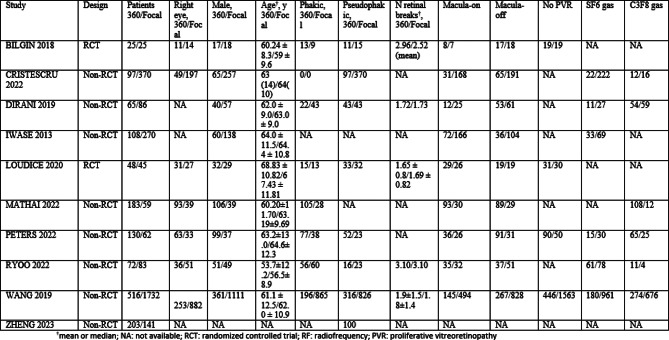
Table with all the baseline characteristics in the studies

### Pooled analyses of all the studies

**Fig. 2:**
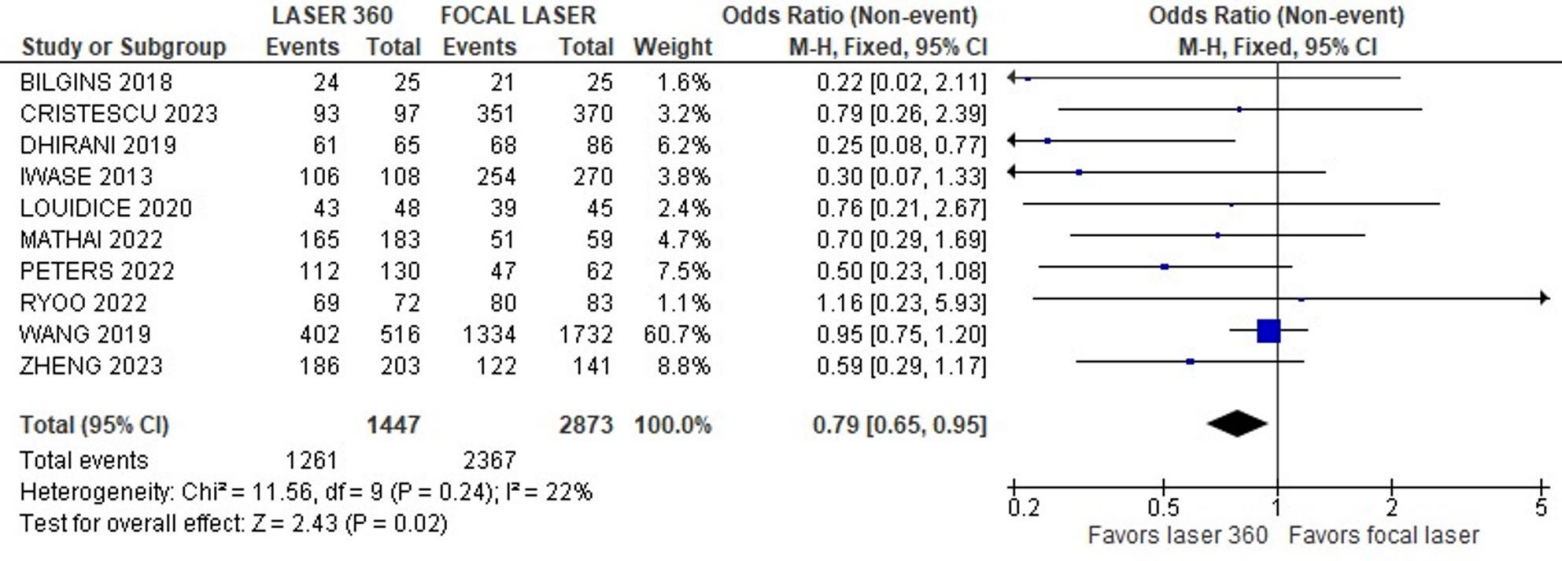
Forrest plot with ten studies that compare SSAS in patients with or without 360 laser. Single surgery success rate

**Fig. 3:**
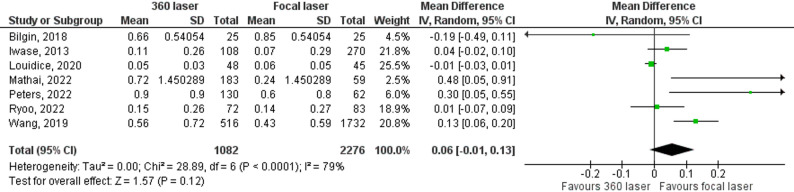
Forrest plot with seven studies that compare BCVA in patients with or without 360 laser. Best corrected visual acuity (BCVA)

We recorded the primary efficacy outcomes of single surgery anatomical success (SSAS) and best-corrected visual acuity (BCVA) as outcomes in ten and six studies, respectively. The pooled analysis of ten articles revealed that the 360-degree laser group was associated with a higher incidence of SSAS (OR = 0.79; 95% Cl 0.65–0.95; Fig. 2). In contrast, the combined analysis of six articles revealed that there was no statistically significant difference in BCVA between the groups (MD = 0.06; 95% CI -0,01–0.13; Fig. 3).

**Fig. 4:**
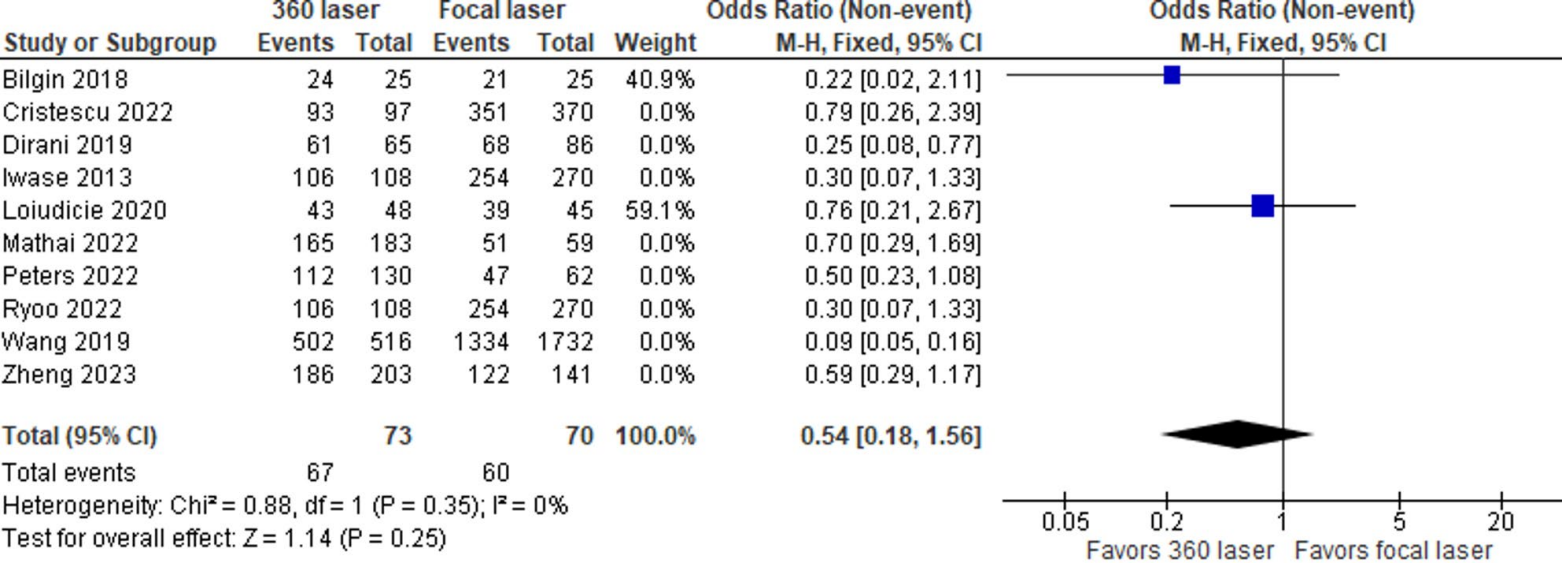
Forrest plot with two studies (only RCTs) that compare SSAS in patients with or without 360 laser. Single surgery success rate (With only RCTs)

When using only the two randomized studies, we continued to see results favorable to the 360 ​​laser, however, now with results lacking statistical significance, and with a fragility index of zero (OR = 0.54; 95% Cl 0.18–1.56; Fig. 4. Table 5) [13].

**Table 5:**
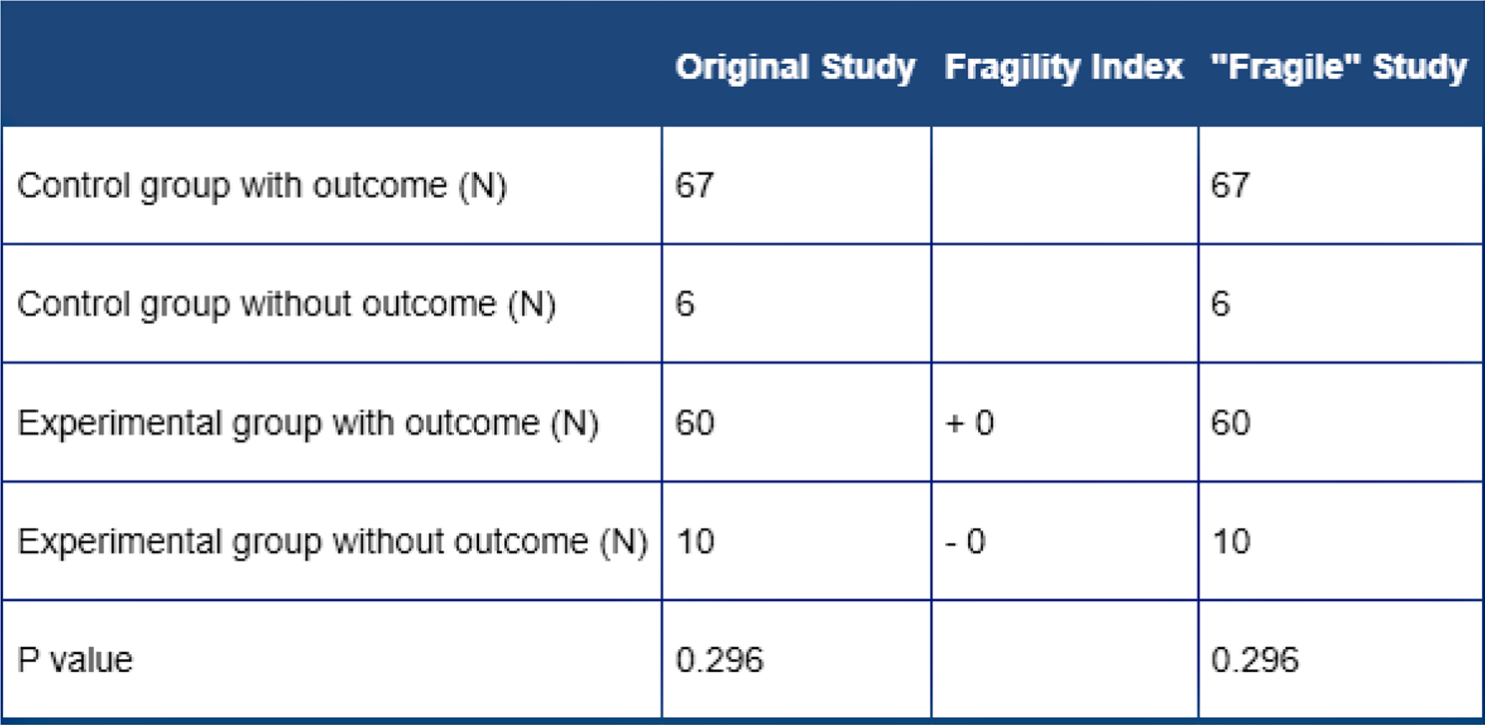
“Fragility Index” is a table used primarily in randomized controlled trials (RCTs) in medical and clinical research, to assess the robustness (or stability) of a statistically significant finding. Fragility Index (ClinCalc.com)

**Fig. 5:**
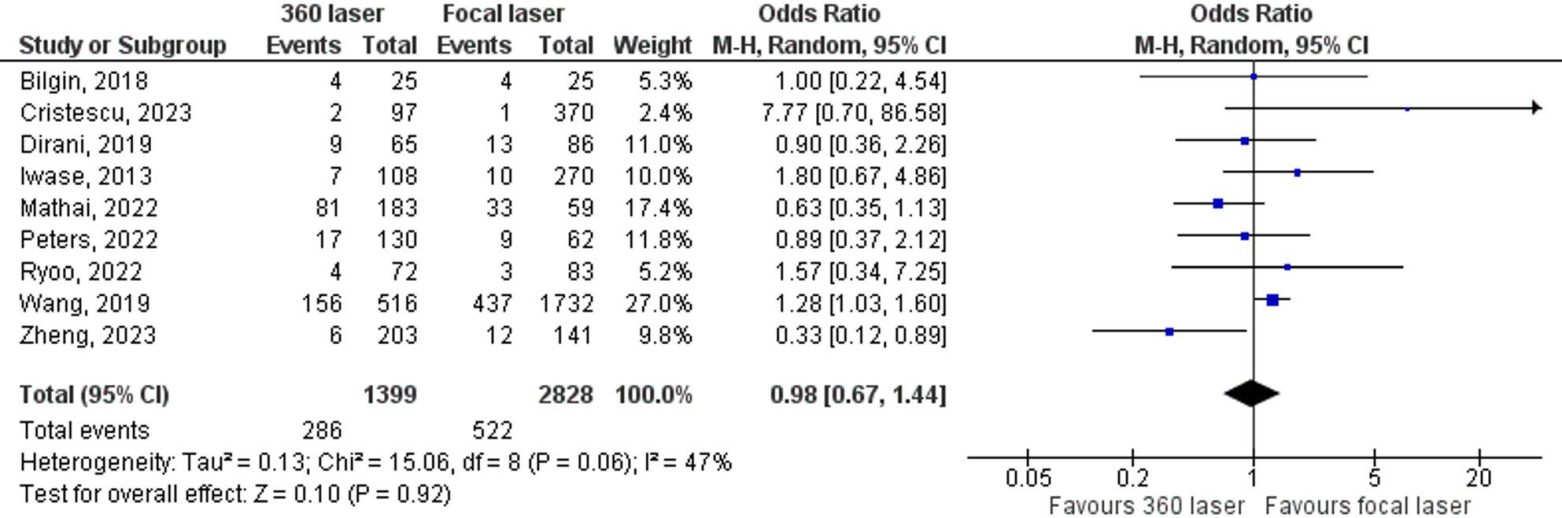
Forrest plot with eight studies that compare ERM formation in patients with or without 360 laser. ERM Formation

**Fig. 6:**
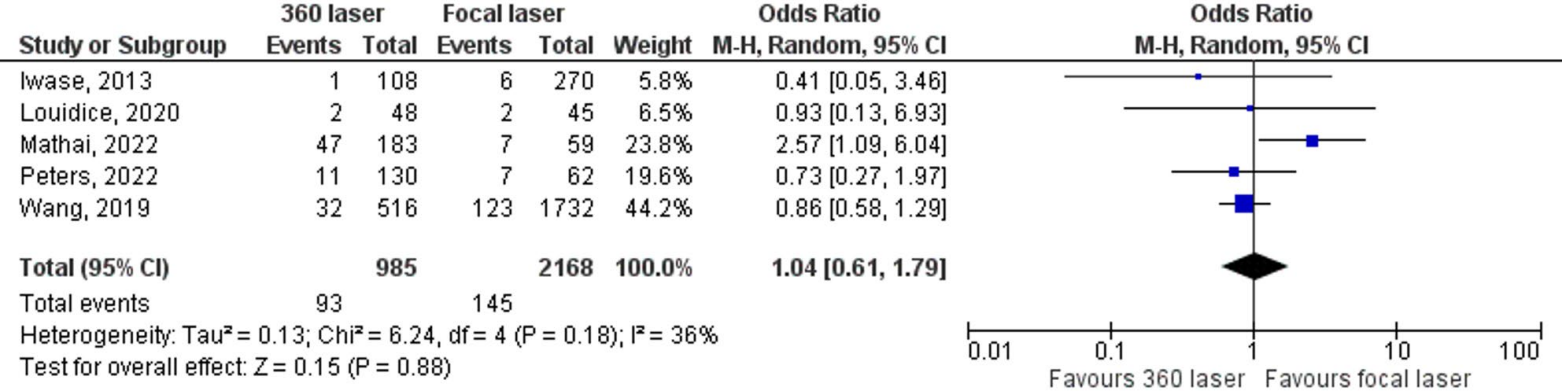
Forrest plot with five studies that compare macular edema in patients with or without 360 laser. Macular edema

We combined nine studies to assess the incidence of epiretinal membrane formation (ERM). The combined analysis revealed a difference in this outcome between the groups, with a lower rate of postoperative ERM development in the focal laser group (OR = 0.98, 95% CI 0.67–1.44; Fig. 5). Finally, the combined analysis of the five studies revealed no significant difference in the rate of macular edema between the groups (OR = 1.04; 95% CI 0.61–1.79; Fig. 6).

### Quality assessment

## Discussion

Our results indicate that RRD repair with a prophylactic intraoperative 360-degree laser might have a higher anatomical success rate for PPV than a focal laser alone. This finding is of special interest, as despite most of the included studies showing a trend in that direction, most of the literature was unable to demonstrate it statistically.

This result aligns with a previous meta-analysis performed by Soekamto et al. in 2021; however, that study had a much smaller pool of patients, and the prophylactic 360 laser had greater anatomical success only when the 23 gauge PPV was analyzed separately [9]. 

Additionally, this previous meta-analysis included a study that compared PPV with a 360-laser versus a scleral buckle alone. This study was not included in our data since we considered the control group to be too different from those in the included studies [9]. 

There is some concern about complications related to the use of extensive laser treatment during vitrectomy. Szigato et al. reported a greater risk of developing epiretinal membranes in eyes that underwent PPV for RRD repair and received more than 1000 laser spots, as did those that received 360-degree treatment [14]. Cakir et al. described a similar result in eyes that received more than 750 treatment locations [15]. In our analysis, compared with the focal laser group, the 360-laser group was associated with more ERM formation, which is consistent with the published literature (OR = 0.98, 95% CI 0.67–1.44).

With respect to functional outcomes, we did not observe any significant difference in best-corrected visual acuity. Interestingly, there is a trend in the direction that favors the focal laser only (MD = 0.06; 95% CI -0,01–0.13).

We acknowledge that our study has several limitations. We included multiple retrospective studies, some of which were not randomized. It is possible that surgeons would prefer 360 lasers in a specific set of patients, creating differences between the groups. Wang et al. performed a multicentric study with 61 surgeons and reported that the use of a 360-laser was more common in younger patients, with larger areas of detachment and a greater number of retinal tears. In addition, it is highly dependent on the surgeon’s preference, as some surgeons perform the procedure much more often [16]. Additionally, our meta-analysis is greatly influenced by the largest study included, Wang et al. [16]. This study has great weight, especially in terms of anatomical success analysis, and is not randomized [16]. 

Although the heterogeneity for our primary outcome (SSAS) remained low (I² = 22%), we acknowledged and investigated the potential influence of study design through a pre-specified subgroup analysis, as detailed in the results. The high heterogeneity observed in the BCVA outcome (I² = 79%) likely reflects the significant confounding and selection bias inherent in the nonrandomized retrospective cohorts, where surgeons selectively applied the 360-degree laser to more complex cases. These study design factors, which we evaluated extensively using the ROBINS-I tool, represent the primary sources of heterogeneity and bias in our pooled estimate for SSAS.

Among the randomized articles, Louidice et al. conducted good randomization intraoperatively, with only the lead surgeon and the assistant aware of which technique would be used. The other examiners were unaware of their choice during subsequent visits [17]. The article by Bilgin et al., on the other hand, used a “pseudo randomization” method, assigning the patients sequentially based on the order they presented. However, the other bias risk criterion in the analysis for both articles were described as “low concern” in the ROB2 assessment tool. Both studies used objective criteria to assess outcomes such as the presence or absence of retinal detachment and employed the standard table for visual acuity (ETDRS) [18]. 

In the nonrandomized studies, there was significant confounding bias in all cases. Mathai et al. conducted a chart review of 241 patients who underwent pars plana vitrectomy between April 2016 and December 2018. They divided the patients into two groups based on whether the surgeons performed a 360° laser or focal laser intraoperatively [19]. Surgeons who made intraoperative decisions were excluded, minimizing bias. Dirani et al. also addressed selection bias through a retrospective chart review from July 2013–July 2016, with surgeries performed by five different surgeons, each using their preferred technique, rather than deciding intraoperatively [8]. Iwase et al. analyzed two prior cohort studies, one from June 2001 to December 2004 with a prophylactic 360° laser and another from January 2005 to September 2007 with only a focal laser, all of which were performed by the same surgeon [20]. 

The remaining five articles, however, were not as rigorous in their indications. The studies by Cristescu et al., Wang et al., Ryoo et al., Peters et al., and Zheng et al. allowed surgeons to make decisions on a case-by-case basis. As a result, more severe cases were treated with a 360° laser, whereas moderate and mild cases received only a focal laser, introducing bias into the final analysis [7, 16, 21–23]. 

Despite this, the results were consistent with the literature, showing low heterogeneity, a considerable sample size, and a validated odds ratio favoring the use of a 360° laser in cases of uncomplicated rhegmatogenous retinal detachment. The analysis of macular edema did not reveal an increased incidence in the postoperative period with the use of a 360° laser (OR 1.04 CI 0.61–1.79). However, when we compared the two groups in terms of best-corrected visual acuity in the postoperative period, the group that received only the focal laser had better results, although the difference was not statistically significant.

This meta-analysis stands out due to its high statistical power (resulting from the robust number of participants/studies) and the application of rigorous methodologies (such as using the ROBINS-I/Cochrane tools and the GRADE system) in evaluating the evidence.

In conclusion, 360° criterion laser prophylaxis during PPV in a single surgery was superior in terms of anatomical success compared with a focal laser in the treatment of RRD. However, these conclusions must be regarded with caution due to the variety of study designs included in this meta-analysis. Combining different study designs, such as randomized controlled trials (RCTs) and retrospective observational studies, offers a broader evidence base, but this approach introduces a risk of selection bias and requires careful statistical handling. The statistical support’s validity is minimally weakened by concerns observed in the randomization process of one of the two included RCTs and the high weighting of nonrandomized studies.

Despite these limitations, the absence of any statistically significant differences in the development of complications between the methods indicates that the choice of treatment should currently be made according to the individual characteristics of the patient and the clinical situation. Therefore, an intraoperative 360-laser may be a safe technique to increase anatomical success.

However, the trend toward better BCVA in the focal laser group and the higher incidence of ERM raise some concerns. Better-designed Randomized Controlled Trials (RCTs) are urgently needed to better support the observed results and establish a standard of care. This issue, as well as the inclusion of nonrandomized retrospective studies, may need to be addressed when more studies and randomized controlled trials are published on this subject. Additional studies could shed more light on the mechanisms behind these observations and assess their effectiveness in combination with other techniques.

## Electronic supplementary material

Below is the link to the electronic supplementary material.

Supplementary Material 1

## Data Availability

No datasets were generated or analysed during the current study.
