# Supplementary figures and images for: Comparative outcomes of focal laser versus 360-degree laser in vitrectomy for retinal detachment: a systematic review and meta-analysis
Source: Int J Retina Vitreous. 2026 Jan 9;12:27. doi: 10.1186/s40942-025-00790-2 (PMC12874893; doi:10.1186/s40942-025-00790-2)

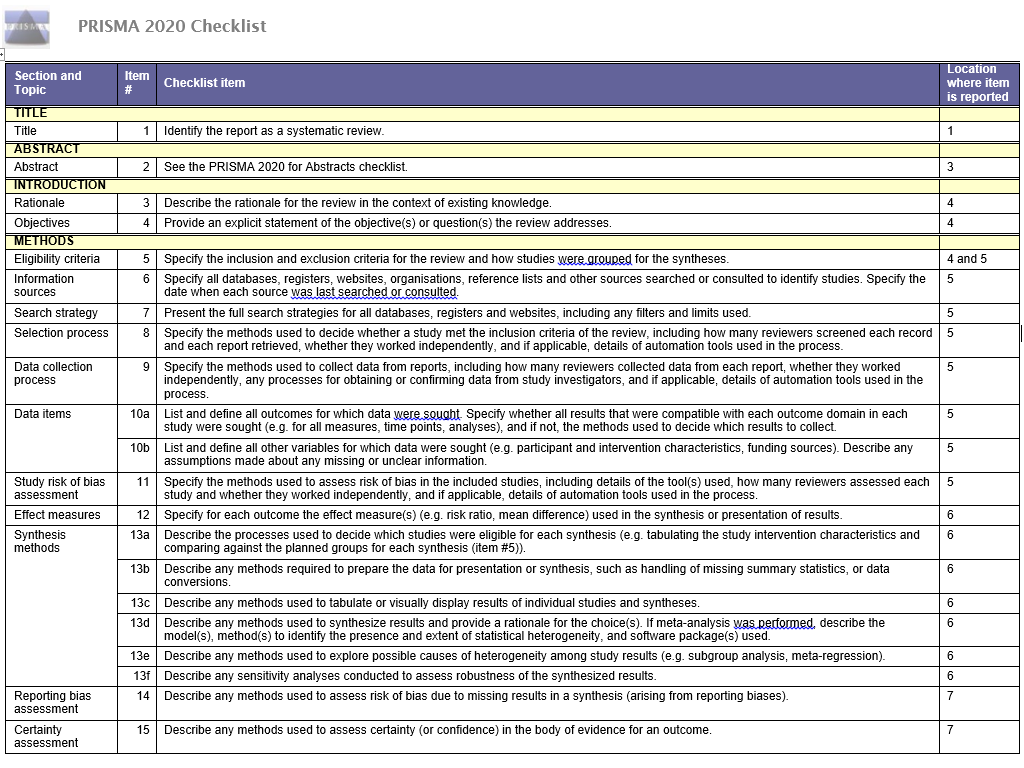

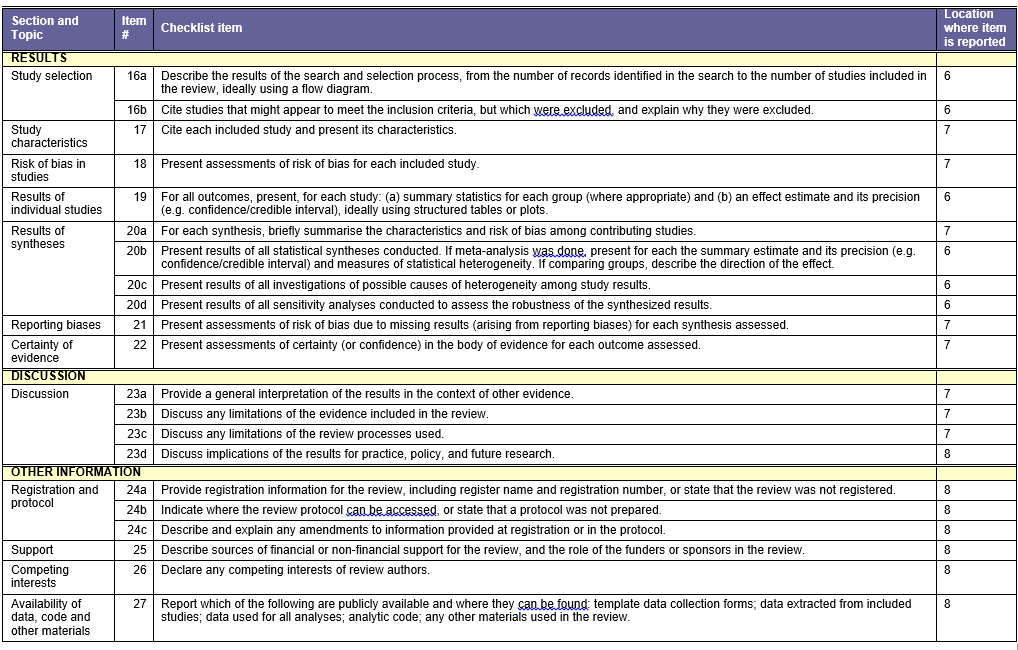

Supplement: Supplementary file 1 — Supplementary Material 1 [file 40942_2025_790_MOESM1_ESM.docx]
